# Supplementary material for: Whole-genome sequence analyses of Glaesserella parasuis isolates reveals extensive genomic variation and diverse antibiotic resistance determinants
Source: PeerJ. 2020 Jun 22;8:e9293. doi: 10.7717/peerj.9293 (PMC7316082; doi:10.7717/peerj.9293)
Supplement: Table S1 — MIC: minimum inhibitory concentration; R: resistant; S: susceptible. a Interpreted according to Clinical and Laboratory Standards Institution (CLSI) guidelines for P. aeruginosa [Clinical and Laboratory Standards Institute. Performance standards for antimicrobial susceptibility testing; twenty-fifth informational supplement. Wayne, PA: CLSI; 2015 Document M100-S25.]. [file peerj-08-9293-s001.docx]

Table S1. Characteristics of *G. parasuis* strain HPS-1, including antimicrobial resistance profile and presence of resistance genes.

| Strain | HPS-1 |
| --- | --- |
| Location | Dongguan |
| Origin | lung |
| Serotype | 4 |
| MLST type | ST 328 |
| Antibiotic resistance profile | Oxacillin-Aztreonam-Amikacin-Gentamicin-Kanamycin-Streptomycin-Ciprofloxacin-Levofloxacin-Trimethoprim/Sulfamethoxazole-Erythromycin-Clairthromycin-Lincomycin-Rifamycin |
| MICs(mg/L) [susceptibility ^a^ ] |  |
| β-Lactams |  |
| Oxacillin | 256 [R] |
| Aztreonam | 2 [R] |
| Imipenem | <0.03125 [S] |
| Meropenem | <0.03125 [S] |
| Ampicillin | 0.75 [S] |
| Cefoxitin | 0.25 [S] |
| Aminoglycosides |  |
| Amikacin | 64 [R] |
| Gentamicin | 256 [R] |
| Kanamycin | 128 [R] |
| Streptomycin | >256 [R] |
| Quinolones |  |
| Ciprofloxacin | 12 [R] |
| Levofloxacin | 16 [R] |
| Sulfanilamides |  |
| Trimethoprim-Sulfamethoxazole | 128 [R] |
| Chloramphenicols |  |
| Chloramphenicol | 1 [S] |
| Tetracyclines |  |
| Tetracycline | 2 [S] |
| Macrolides |  |
| Erythromycin | 256 [R] |
| Clairthromycin | >256 [R] |
| Lincomycin |  |
| Lincomycin | 256 [R] |
| Ansamycin |  |
| Rifamycin | 256[R] |
| Antibiotic resistance genes | *bla _ROB-1_, aac(6')-Ie-aph(2'')-Ia, aph(3'')-Ib, bcr, acrB, sul2* |
| Virulence genes | *Wcip4, wecF, wecG, atpA, atpD, dnaK, fliY, hscA, malG, mglC, mreB, nadR, nqrE, nqrF, oadB2, pntB, uhpT, yckI, yckJ, galE, galU, glmM, gmhA, hemB, hemE, igtF, kdsA, lpxA, lpxC, manB, pgi, rfaD, rfaE, tbpA* |

MIC: minimum inhibitory concentration; R: resistant; S: susceptible.

^a^ Interpreted according to Clinical and Laboratory Standards Institution (CLSI) guidelines for *P. aeruginosa* [Clinical and Laboratory Standards Institute. Performance standards for antimicrobial susceptibility testing; twenty-fifth informational supplement. Wayne, PA: CLSI; 2015 Document M100-S25.].
