# Supplementary material for: Whole-genome sequence analyses of Glaesserella parasuis isolates reveals extensive genomic variation and diverse antibiotic resistance determinants
Source: PeerJ. 2020 Jun 22;8:e9293. doi: 10.7717/peerj.9293 (PMC7316082; doi:10.7717/peerj.9293)
Supplement: Table S2 — N.A. represents unknown information; ST: sequence type; UT: untypeable. [file peerj-08-9293-s002.docx]

Table S2 Sequenced *G. parasuis* genomes involved in this study

| **Isolate** | **Country** | **Source** | **Year** | **Serotype** | **ST** | **Accessiom no.** |
| --- | --- | --- | --- | --- | --- | --- |
| 131 | Japan | Sus domesticus(nasal cavity) | N.A. | 4 | 114 | NZ_MNAL00000000 |
| 16 | China | Sus domesticus(lung) | 2009 | N.A. | 161 | NZ_MNAV00000000 |
| CL120103 | China | swine(heart blood, lungs, ascites, articular, fluid, brain tissue samples | 2012 | N.A. | 320 | [NZ_CP020085](https://www.ncbi.nlm.nih.gov/nuccore/NZ_CP020085.1) |
| F9 | Spain | pig(nose) | 2006 | N.A. | 30 | NZ_JHQI00000000 |
| H12 | China | Sus domesticus(lymphaden) | 2007 | 14 | 168 | NZ_MNAP00000000 |
| H35 | China | Sus domesticus(biood) | 2008 | 15 | 181 | NZ_MNAQ00000000 |
| H36 | China | Sus domesticus(articulation) | 2008 | 14 | 181 | NZ_MNAR00000000 |
| H38 | China | Sus domesticus(lung) | 2008 | 10 | 160 | NZ_MNAW00000000 |
| H39 | China | Sus domesticus(lymphaden) | 2008 | 10 | 160 | NZ_MNAX00000000 |
| H47 | China | Sus domesticus(brain) | 2008 | N.A. | 184 | NZ_MNAS00000000 |
| HPS4 | China | Sus domesticus(lung) | 2009 | 2 | 161 | NZ_MNAT00000000 |
| HPS6 | China | Sus domesticus(lung) | 2009 | 1 | 178 | NZ_MNAZ00000000 |
| K3 | China | Sus domesticus(lung) | 2009 | N.A. | 160 | NZ_MNAY00000000 |
| KL0318 | China | swine(lung) | 2013 | N.A. | 167 | [NZ_CP009237](https://www.ncbi.nlm.nih.gov/nuccore/NZ_CP009237.1) |
| SC1401 | China | swine(lung) | 2014 | N.A. | 346 | [NZ_CP015099](https://www.ncbi.nlm.nih.gov/nuccore/NZ_CP015099.1) |
| SH03 | China | swine(lung) | 2013 | N.A. | UT | NZ_CP009158 |
| YT | China | Sus domesticus | 2008 | N.A. | 161 | NZ_MNAU00000000 |
| SH0165 | China | swine(a Glasser's disease outbreak farm) | 2001 | 5 | 31 | CP001321 |
| 84-17975 | USA | N.A. | N.A. | N.A. | 170 | NZ_AZQU00000000 |
| 29755 | USA | Sus scrofa domestica(lung) | N.A. | 5 | 8 | [NZ_CP021644](https://www.ncbi.nlm.nih.gov/nuccore/NZ_CP021644.1) |
| Nagasaki | Japan | pig(blood of an infected pig with septicemia associated with meningitis)/Sus scrofa domestica | 1986 | 5 | 24 | NZ_APBT00000000 |
| SW114 | Japan | Sus scrofa domestica(nose) | 1986 | 3 | UT | NZ_APBU00000000 |
| MN-H | USA | Sus scrofa domestica(joint; lung; CNS) | N.A. | 13 | UT | NZ_APBV00000000 |
| 12939 | USA | Sus scrofa domestica | N.A. | N.A. | UT | NZ_APBW00000000 |
| 84-15995 | USA | Sus scrofa domestica(lung) | 1992 | 15 | UT | NZ_APBX00000000 |
| H465 | Germany | Sus scrofa domestica(trachea) | 1992 | 11 | UT | NZ_APBY00000000 |
| D74 | Sweden | Sus scrofa domestica | 1992 | 9 | UT | NZ_APBZ00000000 |
| gx033 | China | piglet(lung of a diseased piglet) | N.A. | 4 | 321 | NZ_AOSU00000000 |
| SH0104 | China | pig(lung) | 2016 | N.A. | 347 | [NZ_CP024412](https://www.ncbi.nlm.nih.gov/nuccore/NZ_CP024412.1) |
| HPS10 | USA | pig(lung) | N.A. | 4 | 344 | NZ_JDSO00000000 |
| HPS11 | USA | pig(lung) | N.A. | 4 | 345 | NZ_JDSP00000000 |
| HPS9 | USA | pig(lung) | N.A. | 4 | UT | NZ_JDSN00000000 |
| ST4-1 | USA | pig(lung) | 2011 | 4 | UT | NZ_JJNQ00000000 |
| ST4-2 | USA | pig(lung) | 2012 | 4 | UT | NZ_JJNR00000000 |
| Hp 100/13 | Brazil | swine(lung) | 2013 | 4 | UT | NZ_MOLW00000000 |
| CCUG3712 | UK | N.A. | 1934 | N.A. | 16 | NZ_MUXW00000000 |
| ZJ0906 | China | N.A. | N.A. | 12 | 19 | CP005384 |
| 174 | Switzerland | Sus scrofa domestica(nose) | 1992 | 7 | UT | APCA01000013 |
| SW140 | Japan | Sus scrofa domestica(nose) | 1986 | 2 | UT | APCB01000037 |
| H19 | China | heart blood | 2007 | 7 | 159 | [WBIV00000000](https://www.ncbi.nlm.nih.gov/nuccore/WBIV00000000) |
| H25 | China | Groin Effusion | 2008 | 4 | 164 | WCJO00000000 |
| H26 | China | heart blood | 2008 | 12 | 171 | WAHR00000000 |
| H27 | China | joint fluid | 2008 | 12 | 180 | WCKD00000000 |
| H33 | China | heart blood | 2008 | 4 | 181 | WCKK00000000 |
| H40 | China | lung | N.A. | 4 | 169 | WCKL00000000 |
| H43 | China | head | 2008 | 4 | 176 | WCJI00000000 |
| H45 | China | pericardial effusion | 2008 | 5 | 175 | WCJJ00000000 |
| H46 | China | pericardial effusion | 2008 | 5 | 175 | WCJK00000000 |
| H49 | China | body fluid | 2008 | 10 | 185 | WCJL00000000 |
| H52 | China | lung | 2008 | 1 | 354 | WCJP00000000 |
| H60 | China | spleen | 2008 | 7 | 225 | WCJQ00000000 |
| H61 | China | lung | 2008 | 8 | 355 | WCJR00000000 |
| H64 | China | heart | 2009 | 2 | 356 | WCJN00000000 |
| H68 | China | spleen | 2010 | UT | 176 | WCJX00000000 |
| H74 | China | liver | 2010 | 5 | 341 | WCJV00000000 |
| H78 | China | spleen | 2010 | UT | 342 | WCJU00000000 |
| H80 | China | lung | 2010 | 7 | 342 | WCJT00000000 |
| H82 | China | heart | 2010 | 5 | 343 | WCJZ00000000 |
| H87 | China | pleural effusion | 2010 | 1 | 357 | WCKA00000000 |
| H90 | China | spleen | 2010 | UT | 359 | WCKA00000000 |
| H92 | China | lung | 2010 | 5 | 343 | WCKC00000000 |
| H100 | China | pleural effusion | 2011 | 13 | 322 | WBIW00000000 |
| H105 | China | pericardial effusion | 2011 | 4 | 323 | WBSS00000000 |
| H106 | China | heart | 2011 | 13 | 324 | WBST00000000 |
| H110 | China | ascites | 2011 | 7 | 319 | WBSU00000000 |
| H112 | China | liver | 2012 | 13 | 325 | WBSV00000000 |
| H115 | China | lung | 2012 | 14 | 326 | WBSW00000000 |
| H134 | China | joint fluid | 2012 | 13 | 327 | WCKM00000000 |
| H140 | China | pericardial effusion | 2012 | 13 | 329 | WJHK00000000 |
| H143 | China | brain | 2012 | UT | 329 | WIGS00000000 |
| H157 | China | lung | 2013 | 15 | 330 | WIGT00000000 |
| H159 | China | lung | 2013 | 13 | 331 | WIOJ00000000 |
| H160 | China | pericardial effusion | 2013 | 4 | 254 | WIGU00000000 |
| H164 | China | heart | 2013 | UT | 184 | WIGV00000000 |
| H178 | China | lung | 2013 | 15 | 348 | WIUM00000000 |
| H190 | China | laryngeal airway | 2013 | 13 | 176 | WIUO00000000 |
| H191 | China | laryngeal airway | 2013 | 14 | 332 | WIUL00000000 |
| H197 | China | liver | 2013 | 2&1 | 349 | WIUP00000000 |
| H199 | China | lung | 2013 | 2 | 333 | WIUN00000000 |
| H201 | China | heart | 2013 | 7 | 358 | WIUR00000000 |
| H222 | China | pericardial effusion | 2014 | 4 | 350 | WIUQ00000000 |
| H223 | China | heart | 2014 | 10 | 351 | WIUS00000000 |
| H233 | China | lymphaden | 2014 | 7 | 334 | WCJM00000000 |
| H257 | China | heart | 2015 | 14 | 335 | WCJS00000000 |
| H259 | China | lung | 2015 | 5 | 336 | WCJW00000000 |
| H263 | China | heart | 2015 | 5 | 352 | WCJY00000000 |
| H275 | China | heart | 2015 | 4 | 337 | WCKE00000000 |
| H285 | China | liver | 2015 | 14 | 353 | WCKF00000000 |
| H292 | China | lung | 2015 | 14 | 338 | WCKG00000000 |
| H299 | China | liver | 2016 | 14 | 339 | WCKH00000000 |
| H312 | China | spleen | 2016 | UT | 340 | WCKI00000000 |
| H313 | China | Effusion | 2016 | 13 | 360 | WCKJ00000000 |
| HPS-1 | China | respiratory disease，lung | 2017 | 4 | 328 | CP040243 |
| HPS2 | China | pleural effusion | 2017 | 4 | 285 | CP029150 |

N.A., represents unknown information; ST, sequence type; UT, untypeable.
