# Supplementary material for: Whole-genome sequence analyses of Glaesserella parasuis isolates reveals extensive genomic variation and diverse antibiotic resistance determinants
Source: PeerJ. 2020 Jun 22;8:e9293. doi: 10.7717/peerj.9293 (PMC7316082; doi:10.7717/peerj.9293)
Supplement: Table S3 [file peerj-08-9293-s003.docx]

Table S3. Assembly results for 55 *G. parasuis* genomes sequenced in the present study.

| **Isolate** | **Total length (bp) (bp)** | **Sequencing depth (x)** | **Scaffold number** | **N50 length (bp)** | **GC (%)** |
| --- | --- | --- | --- | --- | --- |
| H100 | 274, 533, 600 | 123 | 124 | 44, 004 | 40.05 |
| H105 | 287, 745, 300 | 132 | 127 | 35, 987 | 39.94 |
| H106 | 268, 024, 800 | 121 | 111 | 54, 075 | 39.92 |
| H110 | 282, 038, 400 | 125 | 95 | 57, 083 | 39.97 |
| H112 | 280, 300, 200 | 126 | 107 | 50, 835 | 39.94 |
| H115 | 280, 068, 900 | 128 | 112 | 41, 051 | 39.94 |
| H134 | 268, 086, 900 | 120 | 110 | 52, 500 | 39.95 |
| H137 | 288, 084, 900 | 128 | 122 | 41, 657 | 39.88 |
| H140 | 273, 847, 800 | 126 | 116 | 44, 000 | 39.99 |
| H143 | 289, 561, 800 | 131 | 130 | 43, 877 | 39.83 |
| H157 | 255, 701, 700 | 103 | 118 | 64, 807 | 39.60 |
| H159 | 268, 483, 200 | 116 | 124 | 49, 250 | 39.98 |
| H160 | 280, 302, 900 | 118 | 128 | 41, 178 | 39.89 |
| H164 | 279, 996, 300 | 129 | 112 | 43, 985 | 39.84 |
| H178 | 292, 894, 800 | 119 | 105 | 63, 377 | 39.62 |
| H19 | 288, 803, 700 | 125 | 96 | 58, 526 | 39.81 |
| H190 | 290, 873, 700 | 133 | 131 | 39, 663 | 39.96 |
| H191 | 289, 251, 900 | 130 | 137 | 39, 866 | 39.83 |
| H197 | 292, 105, 800 | 127 | 165 | 46, 646 | 39.91 |
| H199 | 289, 676, 700 | 130 | 122 | 54, 257 | 40.01 |
| H201 | 290, 422, 200 | 128 | 114 | 42, 967 | 39.80 |
| H222 | 292, 035, 600 | 134 | 122 | 37, 456 | 39.82 |
| H223 | 290, 610, 600 | 119 | 91 | 74, 426 | 39.60 |
| H233 | 286, 950, 600 | 126 | 108 | 61, 120 | 39.89 |
| H25 | 226, 600, 020 | 157 | 30 | 47, 011 | 39.67 |
| H257 | 291, 843, 900 | 132 | 119 | 37, 235 | 39.91 |
| H259 | 292, 238, 700 | 132 | 108 | 47, 719 | 39.93 |
| H26 | 287, 693, 700 | 134 | 133 | 39, 754 | 39.94 |
| H263 | 287, 049, 000 | 133 | 121 | 39, 145 | 39.98 |
| H27 | 290, 609, 100 | 134 | 154 | 35, 256 | 39.75 |
| H275 | 291, 239, 400 | 132 | 134 | 40, 089 | 39.82 |
| H285 | 290, 754, 000 | 136 | 120 | 39, 227 | 39.92 |
| H292 | 289, 974, 000 | 132 | 125 | 40, 199 | 40.03 |
| H299 | 291, 428, 700 | 130 | 134 | 34, 643 | 39.96 |
| H312 | 290, 640, 000 | 128 | 101 | 43, 199 | 39.78 |
| H313 | 289, 903, 800 | 127 | 169 | 36, 712 | 39.93 |
| H33 | 275, 568, 000 | 122 | 171 | 36, 509 | 39.95 |
| H40 | 276, 042, 600 | 125 | 126 | 43, 840 | 39.90 |
| H43 | 287, 615, 400 | 127 | 138 | 37, 538 | 39.88 |
| H45 | 231, 000, 120 | 108 | 131 | 32, 056 | 39.70 |
| H46 | 278, 491, 200 | 129 | 128 | 37, 946 | 39.95 |
| H49 | 287, 681, 400 | 122 | 103 | 81, 649 | 39.71 |
| H52 | 292, 170, 000 | 133 | 120 | 41, 201 | 40.00 |
| H60 | 287, 193, 600 | 134 | 113 | 38, 872 | 39.88 |
| H61 | 260, 165, 700 | 115 | 144 | 39, 513 | 39.99 |
| H64 | 289, 953, 000 | 135 | 111 | 43, 479 | 39.93 |
| H68 | 289, 239, 600 | 135 | 190 | 30, 942 | 40.03 |
| H74 | 286, 561, 500 | 127 | 124 | 36, 926 | 39.87 |
| H78 | 252, 696, 300 | 117 | 121 | 44, 137 | 39.99 |
| H80 | 279, 058, 500 | 129 | 116 | 44, 137 | 40.00 |
| H82 | 279, 564, 000 | 120 | 177 | 37, 820 | 40.04 |
| H87 | 279, 746, 400 | 126 | 87 | 59, 973 | 39.89 |
| H90 | 254, 564, 400 | 115 | 88 | 56, 247 | 39.88 |
| H92 | 275, 161, 500 | 120 | 156 | 37, 342 | 39.97 |
| HPS-1 | 878, 280, 891 | 375 | 1 | 2, 326, 414 | 40.03 |
| HPS-2 | 622, 323, 214 | 275 | 1 | 2, 266, 893 | 40.03 |
