# Supplementary material for: Whole-genome sequence analyses of Glaesserella parasuis isolates reveals extensive genomic variation and diverse antibiotic resistance determinants
Source: PeerJ. 2020 Jun 22;8:e9293. doi: 10.7717/peerj.9293 (PMC7316082; doi:10.7717/peerj.9293)
Supplement: Table S4 [file peerj-08-9293-s004.docx]

Table S4. Orthologous clusters observed in the *G. parasuis* pan-genome.

| **Strain** | **total** | **not_core** | **%Non-core** | **Unique** | **%Unique** |
| --- | --- | --- | --- | --- | --- |
| 12939 | 1, 974 | 1, 780 | 90.17% | 24 | 1.20% |
| 131 | 2, 050 | 1, 854 | 90.44% | 19 | 0.90% |
| 16 | 1, 936 | 1, 739 | 89.82% | 1 | 0.10% |
| 174 | 1, 433 | 1, 252 | 87.37% | 46 | 3.20% |
| 29755 | 2, 182 | 1, 991 | 91.25% | 3 | 0.10% |
| 84-15995 | 2, 206 | 2, 017 | 91.43% | 33 | 1.50% |
| 84-17975 | 2, 061 | 1, 869 | 90.68% | 1 | 0.00% |
| CCUG3712 | 2, 176 | 1, 979 | 90.95% | 11 | 0.50% |
| CL120103 | 1, 962 | 1, 765 | 89.96% | 1 | 0.10% |
| D74 | 2, 218 | 2, 024 | 91.25% | 103 | 4.60% |
| F9 | 2, 318 | 2, 121 | 91.50% | 51 | 2.20% |
| gx033 | 2, 113 | 1, 923 | 91.01% | 10 | 0.50% |
| H12 | 1, 955 | 1, 763 | 90.18% | 3 | 0.20% |
| H35 | 2, 045 | 1, 854 | 90.66% | 0 | 0.00% |
| H36 | 2, 034 | 1, 843 | 90.61% | 0 | 0.00% |
| H38 | 2, 153 | 1, 955 | 90.80% | 0 | 0.00% |
| H39 | 2, 157 | 1, 959 | 90.82% | 0 | 0.00% |
| H465 | 1, 540 | 1, 350 | 87.66% | 32 | 2.10% |
| H47 | 1, 997 | 1, 806 | 90.44% | 8 | 0.40% |
| Hp100-13 | 2, 251 | 2, 044 | 90.80% | 8 | 0.40% |
| HPS10 | 2, 081 | 1, 888 | 90.73% | 14 | 0.70% |
| HPS11 | 1, 912 | 1, 718 | 89.85% | 14 | 0.70% |
| HPS4 | 1, 973 | 1, 777 | 90.07% | 0 | 0.00% |
| HPS6 | 1, 932 | 1, 737 | 89.91% | 3 | 0.20% |
| HPS9 | 2, 060 | 1, 870 | 90.78% | 10 | 0.50% |
| K3 | 2, 195 | 1, 997 | 90.98% | 3 | 0.10% |
| KL0318 | 1, 997 | 1, 807 | 90.49% | 1 | 0.10% |
| MN-H | 1, 859 | 1, 667 | 89.67% | 46 | 2.50% |
| Nagasaki | 2, 260 | 2, 070 | 91.59% | 18 | 0.80% |
| SC1401 | 1, 997 | 1, 802 | 90.24% | 1 | 0.10% |
| SH0104 | 1, 993 | 1, 802 | 90.42% | 1 | 0.10% |
| SH0165 | 2, 031 | 1, 841 | 90.65% | 18 | 0.90% |
| SH03 | 1, 979 | 1, 790 | 90.45% | 5 | 0.30% |
| ST4-1 | 2, 041 | 1, 851 | 90.69% | 5 | 0.20% |
| ST4-2 | 2, 059 | 1, 870 | 90.82% | 8 | 0.40% |
| SW114 | 1, 947 | 1, 759 | 90.34% | 73 | 3.70% |
| SW140 | 1, 484 | 1, 300 | 87.60% | 41 | 2.80% |
| YT | 1, 925 | 1, 729 | 89.82% | 0 | 0.00% |
| ZJ0906 | 2, 211 | 2, 021 | 91.41% | 42 | 1.90% |
| H100 | 2, 143 | 1, 951 | 91.04% | 7 | 0.30% |
| H105 | 2, 088 | 1, 896 | 90.80% | 5 | 0.20% |
| H106 | 2, 121 | 1, 925 | 90.76% | 3 | 0.10% |
| H110 | 2, 163 | 1, 966 | 90.89% | 3 | 0.10% |
| H112 | 2, 124 | 1, 928 | 90.77% | 2 | 0.10% |
| H115 | 2, 086 | 1, 894 | 90.80% | 2 | 0.10% |
| H134 | 2, 150 | 1, 954 | 90.88% | 0 | 0.00% |
| H140 | 2, 063 | 1, 871 | 90.69% | 2 | 0.10% |
| H143 | 2, 103 | 1, 912 | 90.92% | 2 | 0.10% |
| H157 | 2, 357 | 2, 159 | 91.60% | 20 | 0.80% |
| H159 | 2, 225 | 2, 027 | 91.10% | 17 | 0.80% |
| H160 | 2, 350 | 2, 156 | 91.74% | 23 | 1.00% |
| H164 | 2, 074 | 1, 882 | 90.74% | 6 | 0.30% |
| H178 | 2, 370 | 2, 169 | 91.52% | 45 | 1.90% |
| H190 | 2, 095 | 1, 903 | 90.84% | 4 | 0.20% |
| H191 | 2, 128 | 1, 936 | 90.98% | 2 | 0.10% |
| H197 | 2, 210 | 2, 013 | 91.09% | 18 | 0.80% |
| H199 | 2, 121 | 1, 925 | 90.76% | 11 | 0.50% |
| H19 | 2, 209 | 1, 987 | 89.95% | 6 | 0.30% |
| H201 | 2, 166 | 1, 972 | 91.04% | 8 | 0.40% |
| H222 | 2, 056 | 1, 863 | 90.61% | 11 | 0.50% |
| H223 | 2, 321 | 2, 120 | 91.34% | 17 | 0.70% |
| H233 | 2, 207 | 2, 010 | 91.07% | 15 | 0.70% |
| H257 | 2, 139 | 1, 948 | 91.07% | 8 | 0.40% |
| H259 | 2, 118 | 1, 926 | 90.93% | 2 | 0.10% |
| H25 | 1, 231 | 1, 048 | 85.13% | 16 | 1.30% |
| H263 | 2, 049 | 1, 857 | 90.63% | 2 | 0.10% |
| H26 | 2, 036 | 1, 845 | 90.62% | 17 | 0.80% |
| H275 | 2, 133 | 1, 942 | 91.05% | 6 | 0.30% |
| H27 | 2, 046 | 1, 853 | 90.57% | 5 | 0.20% |
| H285 | 2, 044 | 1, 851 | 90.56% | 5 | 0.20% |
| H292 | 2, 109 | 1, 917 | 90.90% | 19 | 0.90% |
| H299 | 2, 174 | 1, 983 | 91.21% | 11 | 0.50% |
| H312 | 2, 180 | 1, 986 | 91.10% | 2 | 0.10% |
| H313 | 2, 193 | 2, 001 | 91.24% | 5 | 0.20% |
| H33 | 2, 166 | 1, 975 | 91.18% | 2 | 0.10% |
| H40 | 2, 124 | 1, 932 | 90.96% | 6 | 0.30% |
| H43 | 2, 162 | 1, 946 | 90.01% | 3 | 0.10% |
| H45 | 2, 015 | 1, 824 | 90.52% | 9 | 0.40% |
| H46 | 2, 079 | 1, 887 | 90.76% | 3 | 0.10% |
| H49 | 2, 256 | 2, 060 | 91.31% | 34 | 1.50% |
| H52 | 2, 092 | 1, 899 | 90.77% | 7 | 0.30% |
| H60 | 2, 029 | 1, 838 | 90.59% | 6 | 0.30% |
| H61 | 2, 270 | 2, 079 | 91.59% | 16 | 0.70% |
| H64 | 2, 047 | 1, 851 | 90.43% | 3 | 0.10% |
| H68 | 2, 044 | 1, 852 | 90.61% | 5 | 0.20% |
| H74 | 2, 187 | 1, 995 | 91.22% | 7 | 0.30% |
| H78 | 2, 072 | 1, 879 | 90.69% | 0 | 0.00% |
| H80 | 2, 079 | 1, 886 | 90.72% | 3 | 0.10% |
| H82 | 2, 248 | 2, 056 | 91.46% | 2 | 0.10% |
| H87 | 2, 140 | 1, 944 | 90.84% | 0 | 0.00% |
| H90 | 2, 138 | 1, 942 | 90.83% | 0 | 0.00% |
| H92 | 2, 227 | 2, 035 | 91.38% | 3 | 0.10% |
| HPS-1 | 2, 288 | 2, 097 | 91.65% | 27 | 1.20% |
| HPS-2 | 2, 336 | 2, 145 | 91.82% | 37 | 1.60% |
