# Supplementary material for: Whole-genome sequence analyses of Glaesserella parasuis isolates reveals extensive genomic variation and diverse antibiotic resistance determinants
Source: PeerJ. 2020 Jun 22;8:e9293. doi: 10.7717/peerj.9293 (PMC7316082; doi:10.7717/peerj.9293)
Supplement: Table S5 [file peerj-08-9293-s005.docx]

Table S5. Characteristics of plasmids compared in this study.

| **Plasmid** | **Country** | **Size (bp)** | **Resistance genes** | **GenBank accession no.** |
| --- | --- | --- | --- | --- |
| pFZ51 | China | 15,672 | *sulII*, *aacC2*, *catAIII*, *parA*, *bla*_ROB-1_, *aph(3’)-I* | JN202624 |
| pFS39 | China | 7,577 | *erm*(T), *bla*_ROB-1_ | KC405064 |
| pHN61 | China | 6,320 | *lnu*(C) | FJ947048 |
| pHB0503 | China | 15,079 | *sulII*, *aacC2*, *catAIII*, *parA*, *bla*_ROB-1,_ *aph(3’)-I* | EU715370 |
| pYL1 | China | 7,777 | *bla*_ROB-1_, *aac(6')-Ie-aph(2'')-Ia* | MK182379 |
