# Supplementary figures and images for: Whole-genome sequence analyses of Glaesserella parasuis isolates reveals extensive genomic variation and diverse antibiotic resistance determinants
Source: PeerJ. 2020 Jun 22;8:e9293. doi: 10.7717/peerj.9293 (PMC7316082; doi:10.7717/peerj.9293)

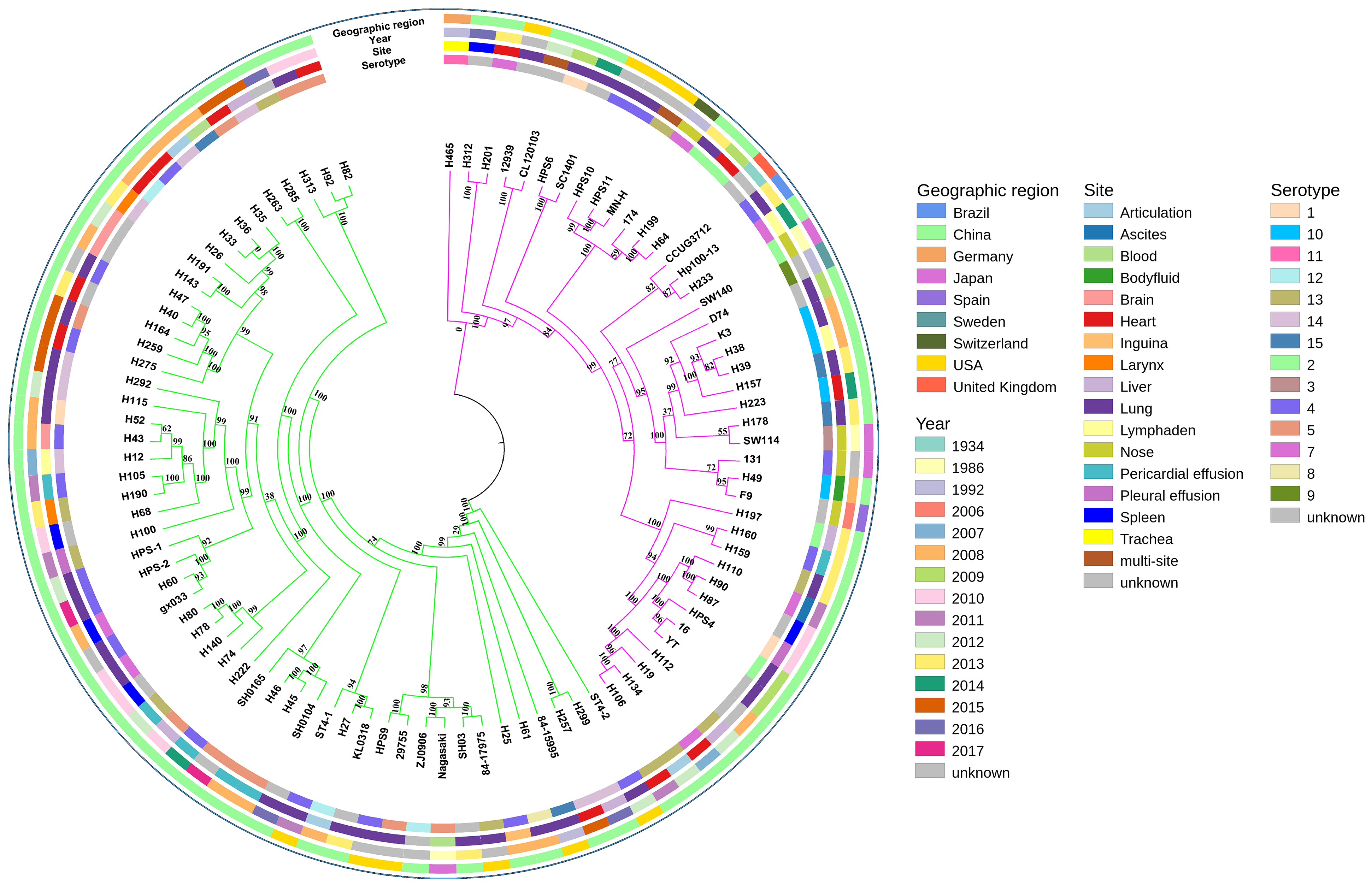

Supplement: Figure S1 — The tree was constructed with MEGA 7 using a maximum-likelihood optimality criterion as implemented in PhyML v3.0 with 1, 000 bootstrap replicates. The annotation rings surrounding the tree, from inside to outside, depict (1) serotype, (2) host, (3) geographic region and (4) year of sample collection. The branch colors denote two major lineages, lineage I (pink) and lineage II (green). [file peerj-08-9293-s006.png]

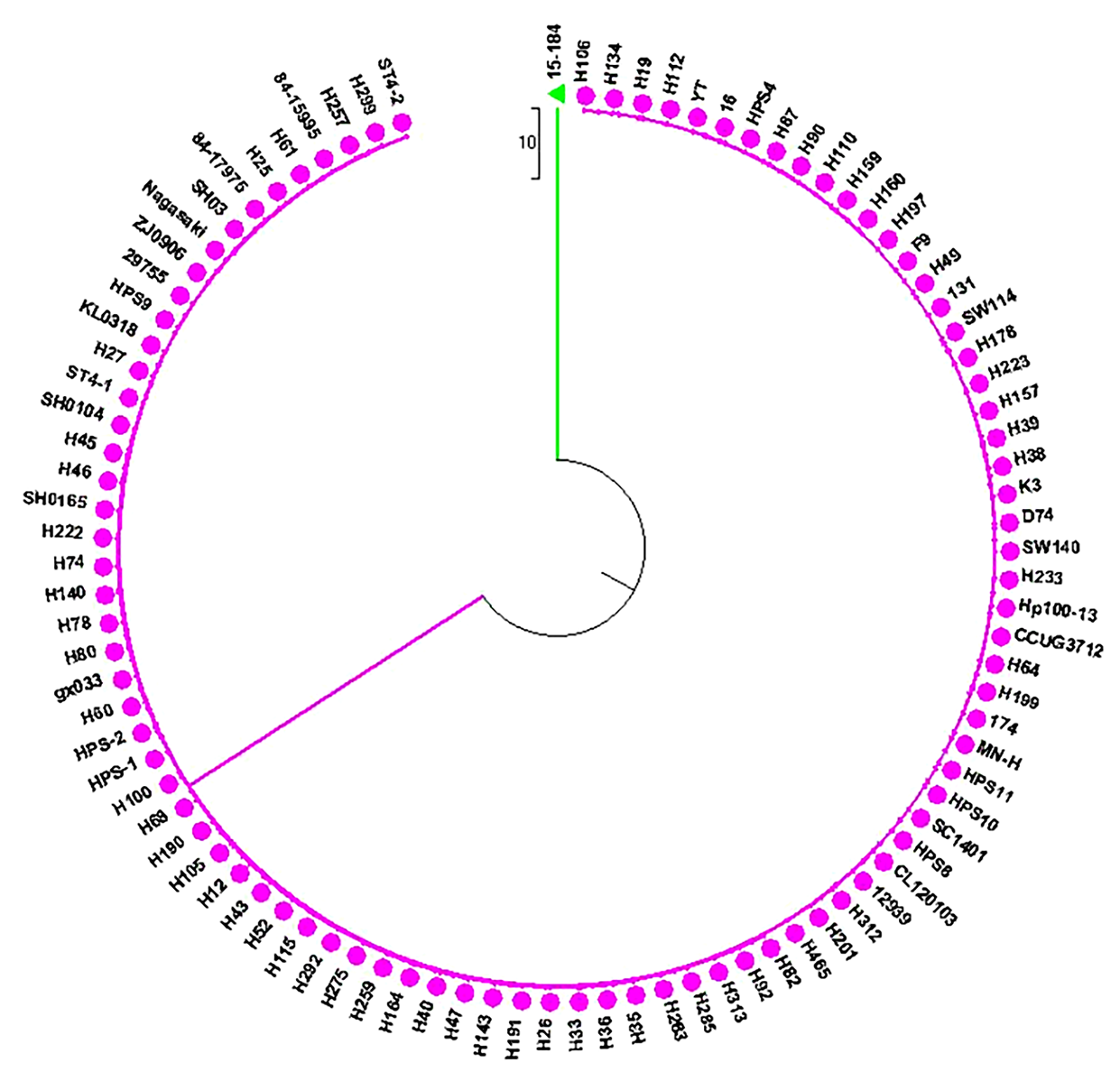

Supplement: Figure S2 — Glaesserella sp. 15–184 was chosen as an outgroup. The branch colors denote two major lineages, lineage I (pink) and lineage II (green). [file peerj-08-9293-s007.png]
