# Supplementary material for: Whole-genome sequence analyses of Glaesserella parasuis isolates reveals extensive genomic variation and diverse antibiotic resistance determinants
Source: PeerJ. 2020 Jun 22;8:e9293. doi: 10.7717/peerj.9293 (PMC7316082; doi:10.7717/peerj.9293)
Supplement: Figure S3 — From inside to outside, the first circle represents the genome size of HPS-1; the second circle represents the GC skew; the third circle represents the GC content; the fourth circle and the seventh circle represent the COG (cluster of orthologous groups) designation of each coding sequence (CDS); the fifth and sixth circles represent the position of CDS, tRNA, and rRNA on the genome. [file peerj-08-9293-s008.pdf]

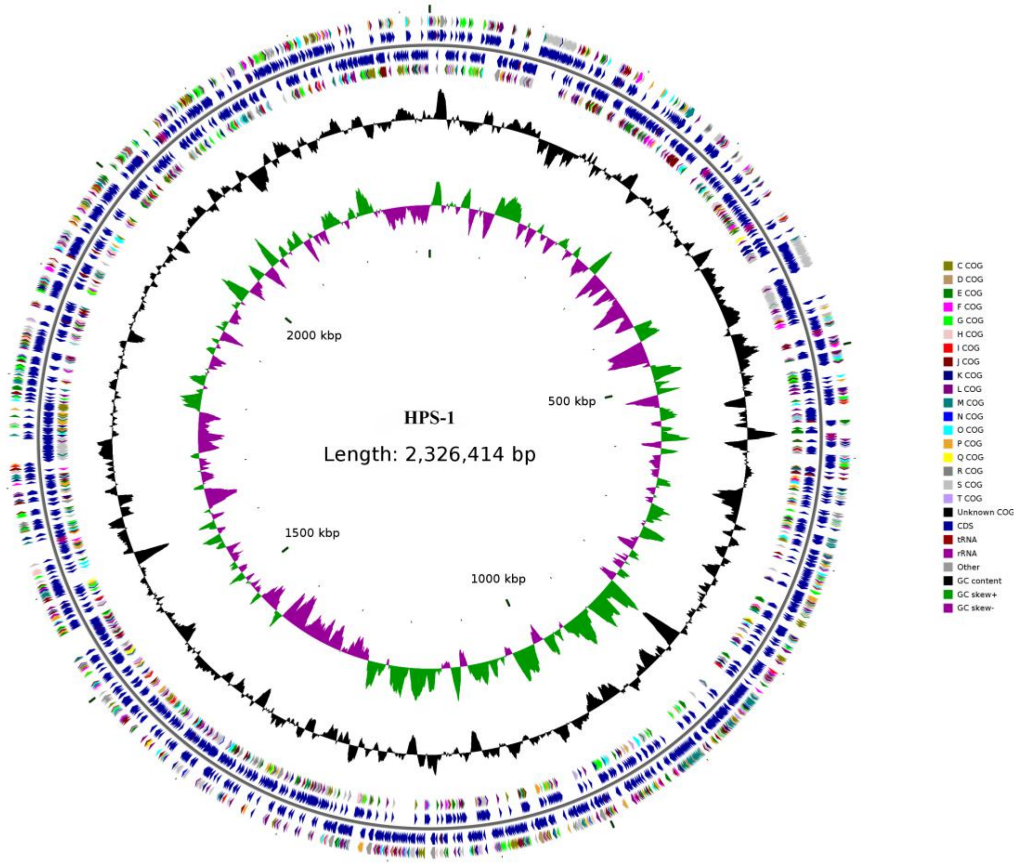

Figure. S3. Circular map of *Glaesserella parasuis* strain HPS-1. From inside to outside, the first circle represents the genome size of HPS-1; the second circle represents the GC skew; the third circle represents the GC content; the fourth circle and the seventh circle represent the COG of each CDS; the fifth and sixth circles represent the position of CDS, tRNA, and rRNA on the genome. CDS; COG; GC; rRNA, ribosomal RNA; tRNA, transfer RNA.
