# Supplementary material for: Whole-genome sequence analyses of Glaesserella parasuis isolates reveals extensive genomic variation and diverse antibiotic resistance determinants
Source: PeerJ. 2020 Jun 22;8:e9293. doi: 10.7717/peerj.9293 (PMC7316082; doi:10.7717/peerj.9293)
Supplement: Supplemental Information 1 [file peerj-08-9293-s009.pdf]

Comparison of antimicrobial resistance and virulence genes

Protein sequences of predicted ORFs were searched for ARGs against the IARDB using BLASTP with an E-value ≤ 10-5. An ORF was designated as an ARG sequence if the best BLASTP alignment to ARG reference sequences was at least 80% similar to a query coverage of at least 70%. Secretory proteins were detected on the genome assembly by SignalP (1). Type I-VII secretion system-related proteins were extracted from all annotation results. Type III secretion system effector proteins were detected by EffectiveT3 (2), and secondary metabolite gene clusters were predicted by antiSMASH (3,4).

References

1. Petersen TN, Brunak S, von Heijne G et al. 2011. SignalP 4.0: discriminating signal peptides from transmembrane regions. Nat Methods 8: 785-6.
2. Jehl MA, Arnold R, Rattei T. 2011. Effective--a database of predicted secreted bacterial proteins. Nucleic Acids Res 39: D591-5.
3. Blin K, Medema MH, Kazempour D et al. 2013. antiSMASH 2.0--a versatile platform for genome mining of secondary metabolite producers. Nucleic Acids Res 41: W204-12.
4. Medema MH, Blin K, Cimermancic P et al. 2011. antiSMASH: rapid identification, annotation and analysis of secondary metabolite biosynthesis gene clusters in bacterial and fungal genome sequences. Nucleic Acids Res 39: W339-46.
