# Supplementary material for: Whole-genome sequence analyses of Glaesserella parasuis isolates reveals extensive genomic variation and diverse antibiotic resistance determinants
Source: PeerJ. 2020 Jun 22;8:e9293. doi: 10.7717/peerj.9293 (PMC7316082; doi:10.7717/peerj.9293)
Supplement: Supplemental Information 2 [file peerj-08-9293-s010.docx]

Abbreviation table

| **Full name** | **Abbreviation** |
| --- | --- |
| *Glaesserella* *parasuis* | *G. parasuis* |
| *Staphylococcus aureus* | 1. *aureus* |
| Multilocus sequence typing | MLST |
| Whole-genome sequencing | WGS |
| Antibiotic resistance gene | ARG |
| Kyoto Encyclopedia of Genes and Genomes | KEGG |
| Clusters of Orthologous Groups | COG |
| Non-Redundant Protein Database databases | NR |
| Gene Ontology | GO |
| Single nucleotide polymorphism | SNP |
| Pathogen Host Interactions | PHI |
| Virulence Factors of Pathogenic Bacteria | VFDB |
| Carbohydrate-Active enZYmes Database | CAZy |
| Integrated Antibiotic Resistance Genes Database | IARDB |
| Brain-heart infusion | BHI |
| Lipopolysaccharide | LPS |
| Major facilitator superfamily | MFS |
| Resistance-Nodulation Division | RND |
